# Role of Natural Bioactive Compounds in the Rise and Fall of Cancers

**DOI:** 10.3390/cancers12092499

**Published:** 2020-09-03

**Authors:** Claudio Luparello

**Affiliations:** Department of Biological, Chemical and Pharmaceutical Sciences and Technologies, University of Palermo, Viale delle Scienze, Edificio 16, 90128 Palermo, Italy; claudio.luparello@unipa.it; Tel.: +39-091-238-97405

Recent years have seen the idea of a close association between nutrition and the modulation of cancer development/progression reinforced. In fact, an increasing number of experimental and epidemiological evidence has been produced, supporting the concept that many different bioactive components of food (e.g., polyphenols, mono- and polyunsaturated fatty acids, methyl-group donors…) may be implicated in either the promotion of or the protection against carcinogenesis. At the cellular level, such compounds can have an impact on different but sometimes intertwined processes, such as growth and differentiation, DNA repair, programmed cell death, and oxidative stress. In addition, compelling evidence is starting to build up of the existence of primary epigenetic targets of dietary compounds, such as oncogenic/oncosuppressor miRNAs or DNA-modifying enzymes, which in turn impair gene expression and function. This editorial aims to summarize the themes of the 31 papers (20 original articles and 11 reviews) published in the Special Issue “Role of Natural Bioactive Compounds in the Rise and Fall of Cancers” presenting the latest findings on the intracellular pathways and mechanisms affected by selected natural molecules influencing the fine-tuning of cancer phenotype.

Plant polyphenols have been among the most studied natural compounds by the contributors to this issue.

In the original article group, Polonio-Alcalà et al. [1] showed the additive and synergistic effects of the flavonoids (−)-epigallocatechin-3-gallate (EGCG) from green tea and its naphthalene derivative G28, which are fatty acid synthase inhibitors, in combination with epidermal growth factor receptor tyrosine kinase inhibitors on gefitinib-resistant lung adenocarcinoma models. Moreover, Wei et al. [2] examined the effect and mechanism of action of EGCG alone and in combination with current chemotherapeutics on pancreatic cancer cell growth, demonstrated the impairment of cell proliferation via the phosphofructokinase inhibition-mediated suppression of glycolysis in a ROS-dependent manner, and the additive enhancement of the anticancer effect of gemcitabine both in vitro and in pancreatic xenografts by the further inhibition of glycolysis and the impairment of cell kinetics. In the Review section, Farooqi et al. [3] analyzed the pleiotropic abilities of EGCG to regulate intracellular signalizations such as those related to JAK/STAT, Wnt/β-catenin, TGF/SMAD, SHH/GLI and NOTCH pathways, also commenting on the ability of EGCG to modulate non-coding RNAs and the methylation-associated machinery in different cancers.

Other natural phenolic compounds whose activity is discussed in the original articles of this issue are:

– Gallic acid (3,4,5-trihydroxybenzoic acid), widely distributed in natural plants, fruits, and green tea, whose tumor-suppressive effect via the p53-mediated downregulation of the transmembrane protein PD-L1 was demonstrated by Kang et al. [4] in non-small-cell lung cancer models;
– Oleuropein, the main bioactive phenolic component of *Olea europaea* L., whose presence in enriched extracts from olive leaves was proven to reduce the glycolytic rate of a wide range of solid and liquid tumor cells via the downregulation of the three key effectors of the glycolytic pathway, GLUT-1, PKM2 and MCT4, likely resulting in a decreased glucose entrance and biomass production [5];
– Oleacein (3,4-dihydroxyphenylethanol), the main secoiridoid contained in extra virgin olive oil, able to elicit significant anti-tumor activity by promoting cell cycle arrest and apoptosis in multiple myeloma cells due to its histone deacetylase inhibitory properties [6];
– Dadzein (7,4′-dihydroxyisoflavone), present in soybeans, whose 4-sulphate metabolite produced by gut microbiota was found to exert an anti-estrogenic effect on ERα-positive breast cancer cells via the downregulation of the anti-apoptotic neuroglobin protein thus rendering cells more prone to the paclitaxel treatment [7];
– Gigantol, a bibenzyl compound from orchid species, whose ability to destabilize tumor integrity via the suppression of the PI3K/AKT/mTOR and JAK/STAT pathways was demonstrated by Losuwannarak et al. [8] in non-small-cell lung cancer models in vitro and in vivo;
– Lonchocarpin, a chalcone isolated from *Lonchocarpus sericeus*, proven to be a powerful inhibitor of the Wnt/β-catenin pathway able to selectively suppress the migration and proliferation of a panel of colorectal cancer cell lines in vitro and in a preclinical colorectal cancer mouse model [9];
– Isorhamnetin, (3′-methoxy-3,4′,5,7-tetrahydroxyflavone), a flavonol aglycone found in some medicinal plants, able to exert an anti-proliferative effect on human bladder cancer cells via the induction of cell cycle arrest during the G2/M phase and apoptosis, accompanied by the activation of the AMPK signaling pathway and ROS overproduction [10];
– Erioquinol, eriopodol A and gibbilimbol B, derived from *Piper* genus plants, whose ability to inhibit XIAP protein, involved in the regulation of caspase-dependent/independent cell death pathways, was reported by Muñoz et al. [11] in breast cancer cell lines;
– Vatein, isolated from *Calocedrus formosana* Florin leaves extract, proven to interfere with cell cycle and microtubule dynamics in lung adenocarcinoma cells, also inhibiting tumor growth in a xenograft mouse model [12].

In the Review section, Perrone et al. [13] discussed the effects of polyphenols in preventing the progression of central and peripheral nervous system tumors underlining the beneficial effect of dietary compounds on the microbioma–intestine–brain axis. Barbosa and Martel [14] examined the role played by a wide variety of synthetic and natural substances, including polyphenols, on the impairment of glucose uptake by neoplastic breast cells thereby resulting in a tumor-restraining effect. Ong et al. [15] reported the broad-range in vitro/in vivo anticancer properties of the *Magnolia*-derived polyphenol honokiol based upon its ability to impair cell cycle progression, inhibit epithelial–mesenchymal transition, and suppress cell motility, invasion, metastasis and angiogenesis. Zhou et al. [16] summarized the late preclinical studies on the applications of bioactive polyphenols in lung cancer therapy, focusing on the molecular mechanisms at the basis of their therapeutic effects and also discussing the potential of the polyphenol-based combination therapy. Goh et al. [17] reviewed data on the anti-colon cancer properties of nobiletin, a polymethoxyflavone extracted from citrus peel, and its derivatives which are able to arrest the cell cycle, inhibit cell proliferation, induce apoptosis, prevent tumor formation, reduce inflammatory effects and limit angiogenesis, also exploring better drug delivery strategies due to the low oral bioavailability of the compounds. Ong et al. [18] focused their review on the pharmacological properties and therapeutic potential of formononetin [7-hydroxy-3-(4-methoxyphenyl)-4H-1-benzopyran-4-one], one of the main bioactive components of red clover, which regulates various transcription factor- and growth factor-mediated oncogenic pathways attenuating metastasis and tumor growth in vivo in multiple cancer cell models and also alleviating the possible causes of chronic inflammation that are linked to the cancer survival of neoplastic cells and their resistance against chemotherapy.

The other articles and reviews addressed further cancer-related issues relevant to types of compounds of a different nature, specifically:

– The methanolic extract of *Malva pseudolavatera* leaves, which showed a promising selective anti-proliferative and pro-apoptotic effect on acute myeloid leukemia cell lines, determining PARP cleavage, cytochrome-c release, Bax/Bcl-2 ratio increase and ROS overproduction [19];
– Eicosapentaenoic acid, an ω-3 polyunsaturated fatty acid, which played a protective role, both alone and in combination with angiotensin-converting enzyme inhibitors, in attenuating adipocyte-induced proinflammatory cytokine expression and the migration of breast cancer cells in an in vitro model of obesity-induced breast cancer [20];
– Fucoidan, a sulphated polysaccharide derived from brown seaweed, whose combination with gemcitabine determined an enhanced pro-apoptotic and cell cycle-inhibitory activity on selected uterine carcinosarcoma and stromal sarcoma cell lines [21];
– Nicotin, whose mechanisms underlying the promotion of melanoma cell proliferation and migration mediated through α9-nAChR-initiated carcinogenic signaling and PD-L1 expression were reported by Nguyen et al. [22];
– The ethyl acetate fraction of the crude extract of *Streptomyces* sp. MUM256, isolated from mangrove soil in Malaysia, and the cyclic dipeptides contained whose ability to induce cell cycle arrest and apoptosis was demonstrated by Tan et al. [23] in colon cancer cells;
– Manoalide, an antibiotic sesterterpenoid isolated from the marine sponge *Luffariella variabilis*, which preferentially inhibits the proliferation of oral cancer cells inducing apoptosis and DNA damages via oxidative stresses, such as intracellular ROS and MitoSOX/MitoMP [24];
– λ-carrageenan, a family of linear sulfated polysaccharides, proven to enhance the effect of radiotherapy by suppressing the survival and invasiveness of different cancer cell lines in vitro and in vivo through the Rac GTPase-activating protein 1 pathway [25];
– Ethanol, which was found to trigger a pro-survival autophagic response following the induction of oxidative and endoplasmic reticulum stress in colon cancer cells, and the activation of Nrf2 and HO-1 also leading to the acquisition of a more aggressive phenotype [26];
– Colchicine, an alkaloid present in the medicinal plant *Colchicum autumnale*, whose enhanced anticancer effects and reduced cytotoxicity on colon cancer cells when delivered in the nanoformulated form was reported by AbouAitah et al. [27].

In the Review section, Del Cornò et al. [28] discussed the modulatory effects of dietary β-glucans, present in diverse edible mushrooms, baker’s yeast, cereals and seaweeds, on human innate immunity cells and their potential role in cancer control. Lee et al. [29] reviewed a large number of data on the role played by different cytokines, lipids and other natural molecules on the suppression of epithelial–mesenchymal transition in cancer progression. Ennour-Idrissi et al. [30] focused their attention on the bioaccumulation of persistent organic pollutants in the food chain and the association of exposure with breast cancer risk. Farooqi et al. [31] presented the current views about the ability of berberine, a natural alkaloid compound found in several medicinal plants, to target different signaling cascades in various cancers, also discussing the nanocarrier strategies developed to improve the delivery of the compound.

The number of manuscripts published in this Special Issue indicates an active interest in research about the molecular/pharmacological mechanisms used by natural products exerting anti-tumoral effects which deserve further and deeper studies. I wish to thank all the contributors of this issue for sharing with us their experimental or reviewed data which will surely attract readers’ attention and encourage the publication of other high-quality papers in this field.
